# Science vs. the Senses

**Published:** 1871-01

**Authors:** 


### SCIENCE *VS.* THE SENSES.

It has always been the office of science to separate that which *is* from that which only *seems.*

On the trial of a mal-practice case in the District Court of Clayton County, Iowa, in September last, an interesting contest arose between "liberal science" and the senses of plain, common people.

The alleged mal-practice was in the treatment of a fracture of both bones of the plaintiff's right forearm, at a point about four inches from the wrist.

The defendant was a "liberal and progressive surgeon," who, after the commencent of the suit, spent a few weeks in Chicago, in connection with a celebrated medical school of that city. It was but natural that the defendant should draw around him other gifted and liberal minds of his profession. Prominent among his new friends, was one R. A. Gunn, M.D., Professor of Surgery, Bennett Medical College, and one of the editors of the *Chicago Medical Times*, who, ever ready to sacrifice personal comfort in behalf of science, volunteered to be present at the trial in person, to assist the persecuted defendant by his testimony as an expert, and by his counsels as a friend.

On the trial, an inquiry was made touching the position of the injured arm during the treatment. The plaintiff, his wife, and some of his neighbors, testified that the hand was nearly prone when the dressing was first applied, and that it remained so during the subsequent treatment.

To rebut this testimony, the learned Professor was called to the witness' stand, who testified, in substance, "that if the hand were prone before the dressing was applied, it could not remain so one moment afterwards; that the ordinary pressure of the bandage required to fasten the splints, would inevitably change the hand from a prone position, to a position midway between pronation and supination, and that this result would follow whether the bones were whole or broken."

To demonstrate this theory to the jury, the learned Professor produced the bones of a man's forearm, the continuity of which had not been disturbed. These he placed in the relations which they assume in the living arm, where the hand is prone, the one crossing the other obliquely and then by a slight pressure near the upper end, the one was made to slip down by the side of the other, leaving them as nearly parallel as the shape of the bones would permit. The learned Professor, then very clearly showed, that in the living subject these bones are only parallel "when the thumb is up," and again drew the conclusion that the stupid witnesses for the plaintiff must be mistaken.

One of the attorneys for the plaintiff, however, unwilling to accept the situation, requested the learned Professor to use the living arm for the purposes of the demonstration, instead of those "naked bones tied together with a string." The proposition was promptly and cheerfully accepted, and for the experiment the learned Professor took the arm of the attorney who made the request. Placing the hand in a prone position, the Professor, applied, instead of pasteboard splints, two books, extending from the wrist nearly to the elbow, and, instead of the roller, he used his hands to give the necessary pressure. To the educated and professional reader it is unnecessary to say, that the hand instantly and "inevitably flopped up" into the desired position, but as the jury, country doctors, and other witnesses of the experiment, were plain, uneducated folks, with no guide but their senses, they *couldn't see it.* To them the position of the hand seemed altogether unchanged. The learned Professor, noticing this defect of their vision, attempted to change the apparent result by increased pressure on the books.

Still failing to change the appearances, he made one more desperate effort in behalf of liberal science, by a violent attempt to turn the hand in the position desired. Failing again, he quit the contest, assuring the jury "that the thing would work" just as he had stated, but the "inevitable mechanical result" had been prevented by the wicked will of the subject whose arm he had used. The learned Professor, of course, knew that the thing *had worked*, and he cunningly made this admission to the contrary, because it was too evident that the ignorant jury could not be made to doubt the testimony of their own eyes, and that the cause of truth, as well as the interests of the defendant, required a solution that would be satisfactory to them. That the learned Professor knew that the difficulty was in their vision, and not in the will of the subject, is evident from the fact, that when it was proposed that the experiment should be repeated on the arm of one of the defendant's own attorneys, or on the arm of one of the jury, the Professor gracefully declined.

It would be difficult to say too much in praise of the course pursued by the Professor during this trial. While the country doctors appeared only in obedience to the imperative process of the Court, and then took back seats, apparently indifferent to the result. The learned Professor not only volunteered to vacate his chair in Chicago, during this long and tedious investigation, but from the beginning to the close, he was seated within the bar, in close proximity to the defendant and his counsel, never failing to manifest his zeal for science by suggesting profound questions to be put to witnesses, by taking notes, and by smiling, frowning, or winking, as the changing circumstances seemed to demand.

Let no friend of "liberal and progressive science" be discouraged in consequence of any temporary defeat. Be patient and labor on. It took courtesies to convince the ignorant masses that the sun does not make a daily journey around the earth.

JUSTICE.

